# Usability Across 3 mHealth Problem-Solving Training Interventions for Diverse Neurodevelopmental and Neurological Populations: Multicase Usability Evaluation

**DOI:** 10.2196/90379

**Published:** 2026-09-09

**Authors:** David Ogundairo, Shannon Juengst, Avani Modi, Shari Wade, Matthew Schmidt

**Affiliations:** 1 Department of Workforce Education and Instructional Technology, Mary Frances Early College of Education, University of Georgia, Rivers Crossing 110, Athens, GA, 30602, United States, 1 7062548087; 2 Brain Health and Rehabilitation Research Center, TIRR Memorial Hermann, Houston, TX, United States; 3 Department of Physical Medicine & Rehabilitation, UT Health Houston, Houston, TX, United States; 4 College of Medicine, University of Cincinnati, Cincinnati, OH, United States; 5 Division of Behavioral Medicine and Clinical Psychology, Cincinnati Children’s Hospital Medical Center, Cincinnati, OH, United States; 6 Division of Physical Medicine and Rehabilitation, Cincinnati Children’s Hospital Medical Center, Cincinnati, OH, United States; 7 Department of Clinical and Administrative Pharmacy, College of Pharmacy, University of Georgia, Athens, GA, United States

**Keywords:** usability, mobile health, psychoeducation, problem solving, digital health intervention, sociotechnical systems, user experience, mobile phone

## Abstract

**Background:**

mHealth (mobile health) interventions that integrate psychoeducation with structured problem-solving training (PST) hold strong potential for improving self-management of chronic conditions. Evaluating the usability of these interventions requires assessing technological, pedagogical, and sociocultural fit. However, most usability evaluations remain narrowly technocentric, focusing on interface-level metrics while neglecting pedagogical coherence, cultural responsiveness, and patient learning needs.

**Objective:**

This exploratory study aimed to characterize usability challenges and facilitators across 3 psychoeducational mHealth PST interventions and to identify technological, pedagogical, and sociocultural design features that can improve engagement, accessibility, and implementation for diverse users.

**Methods:**

This study used an exploratory, multimethod, collective case study design. Three independent mHealth PST usability studies used structured think-aloud protocols, with sessions conducted remotely via Zoom (Zoom Communications, Inc), except for 1 in-person session for Epilepsy Journey 2.0. A total of 14 participants were enrolled across 3 independent cases via purposive sampling from their respective target populations. Case 1 – Epilepsy Journey 2.0 (n=6; 4 male, 2 female; ages 12‐18 y; adolescents with epilepsy), case 2 – Survivor’s Journey (n=3; 1 male, 2 female; ages 18‐30 y; adolescent and young adult survivors of brain tumor), and case 3 – electronic problem-solving training (n=5; 2 male, 3 female; ages 30‐65 y; adults with a history of severe traumatic brain injury). Participants completed a presession technology comfort survey and the Comprehensive Assessment of Usability for Learning Technologies postsession. All sessions were recorded, transcribed, and analyzed thematically. Comprehensive Assessment of Usability for Learning Technologies data were analyzed using descriptive quantitative methods.

**Results:**

Electronic problem-solving training demonstrated the highest usability (mean 87.96, SD 13.73), followed by Survivor’s Journey (mean 83.00, SD 7.70), and then Epilepsy Journey 2.0 (mean 79.00, SD 12.75). Findings revealed that usability in health care learning design is shaped by how effectively the technology, learning content, and contextual factors align with patients’ needs. Recurring challenges across interventions included unclear navigation, poor mobile responsiveness, instructional ambiguity, insufficient feedback, potential for greater inclusivity, and limited error recovery. Twelve cross-case design principles were derived, emphasizing mobile-first accessibility, cognitive load reduction, context-sensitive feedback, and empathetic, inclusive design.

**Conclusions:**

Usability challenges in mHealth PST interventions arise not only from interface-level issues but also from how effectively the intervention supports users’ understanding, decision-making, and real-world application demands. This extends prior mHealth usability research by demonstrating that user difficulties often reflect misalignments between technological features, instructional structure, and the everyday contexts in which individuals engage with PST. The resulting design principles highlight specific, actionable priorities for developers, including mobile-first optimization, clearer task scaffolding, and better feedback and error recovery. Future work should evaluate these principles in larger samples and clinical settings to determine their impact on engagement, adherence, and downstream health outcomes.

## Introduction

### Background

Digital health interventions have become foundational to modern health care, offering accessible, scalable, and patient-centered models of care [1,2]. Among the most promising are mHealth (mobile health) interventions that integrate psychoeducation with structured problem-solving training (PST). Grounded in social-cognitive and self-regulation frameworks, these interventions equip patients not only with disease-specific knowledge but also with step-by-step strategies for identifying barriers, generating solutions, and applying them to real-world health challenges [3-6]. Across diverse populations, PST-based mHealth interventions have demonstrated consistent benefits, including reductions in depressive and anxiety symptoms, improvements in medication adherence, and increases in caregiver self-efficacy [6-8].

### Usability Is Central to the Success of mHealth Interventions

While rigorous, evidence-based design constitutes the methodological bedrock of digital therapeutics, usability, a central dimension of patient experience, is pivotal for uptake and sustained engagement, which in turn mediate clinical effectiveness [9-11]. Usability refers to how effectively, efficiently, and satisfactorily patients can interact with technology to achieve their desired goals [12]. The ISO defines usability as the extent to which a system, product, or service can be used by specified users to achieve a specified goal, encompassing five attributes: effectiveness, efficiency, satisfaction, freedom from risk, and context coverage [13]. Well-designed mHealth interventions that prioritize usability and patients’ experiences are more likely to be adopted, maintained, and found beneficial by patients over time [14]. Conversely, poor usability can lead to frustration, disengagement, and abandonment, directly undermining any clinical benefit the intervention might offer [15]. For example, high-school testers in the LIFE4YOUth study reported that poor functionality and difficult navigation made them cease using the app altogether [16], while a systematic review of 60 studies on electronic personal health records and digital mental health tools consistently identifies poor usability as a primary driver of low adoption and high dropout [17,18]. As PST mHealth interventions are complex, serve diverse patient populations, and play a central role in self-management, comprehensive usability evaluations are increasingly regarded as indispensable for designing effective digital tools that support behavior change, chronic disease management, and mental health outcomes [19,20].

### General Usability Measures Are Limited for Evaluating mHealth Psychoeducation

Despite growing recognition of usability’s importance, most digital health studies rely on general human-computer interaction instruments for usability evaluation [21,22]. The most widely used is the System Usability Scale (SUS) [23], a 10-item questionnaire yielding a composite score from 0 to 100 that captures users’ overall perceptions of ease of use and learnability; scores around 50 are generally considered marginal, scores around 70 are considered good, and scores above 85 are considered excellent [24]. Other common instruments include the Computer System Usability Questionnaire [25] and the Software Usability Measurement Inventory [26]. While these tools can efficiently capture surface-level interface mechanics such as ease of use, interface flaws, and navigation flow, they were not designed to evaluate pedagogical alignment, social dynamics, or cultural responsiveness [22,27]. These dimensions are especially important for psychoeducational mHealth interventions, which share structural features with learning technologies (eg, modularized content, formative assessments and feedback, and self-directed practice) and require more than interface usability to support meaningful health behavior change.

Narrowly focused mHealth psychoeducation usability evaluation presents a significant limitation in light of the layered interactions between content, patient needs, and learning objectives in these interventions. Indeed, evidence shows that strong perceived technological usability does not guarantee meaningful impact. In a diabetes self-management study, participants rated the app high on the SUS (mean 80.5, 95% CI 72.3 to 88.7), yet struggled with key tasks, recording the most errors and lowest success rates on activities essential for effective self-management [28]. A pilot randomized trial of an osteoarthritis physical-activity app likewise found acceptable usability ratings (mean 71.3), but found no significant improvement over usual care for most outcomes [29]. Similarly, a usability study of home blood-pressure and pulse-oximeter devices reported satisfactory SUS scores for both a blood pressure monitor (mean 72.4, 95% CI 68.8 to 76.0) and a pulse oximeter (mean 71.52, 95% CI 68.7 to 74.3), despite users making frequent handling errors and some abandoning the devices altogether [30]. These findings illustrate that high usability ratings can coexist with limited learning, behavior change, or health benefits, suggesting a need for more holistic approaches to usability capable of not only evaluating technological aspects, but also aspects such as patient relevance, learning effectiveness, and social acceptability.

### Addressing Usability Gaps in Health Care Learning Design Through Multidimensional Evaluation

Although more comprehensive evaluation frameworks exist, most usability studies in health care learning remain narrowly focused on interface-level metrics. A growing body of research documents this gap and its consequences. A scoping review of 124 digital health tools for individuals with multiple sclerosis found that only 26% conducted usability testing, and among those, the majority used limited metrics [31]. A review of sensor-based health technologies found that while 83%‐91% assessed usability through satisfaction measures, fewer than 7% evaluated efficiency, and only 9% addressed users’ ability to interpret clinical data [32]. A broader analysis of 610 digital health usability studies found that nearly half relied solely on the SUS, with little to no attention to accessibility, instructional utility, or cultural fit [33]. Minimal consideration of contextual or educational dimensions has similarly been noted in health IT evaluations [34] and in health care education specifically, where most mobile app usability studies relied on checklists and surveys, rarely assessing whether tools effectively supported learning or improved patient understanding [35]. The consistent absence of pedagogical and contextual considerations in usability evaluations may explain the high abandonment rates observed for health-related digital tools [36-39], and highlights the necessity of frameworks capable of capturing the complex and multifaceted nature of health care learning environments.

### Addressing Usability Gaps in Health Care Learning Design Through Multidimensional Evaluation

**Figure 1.**
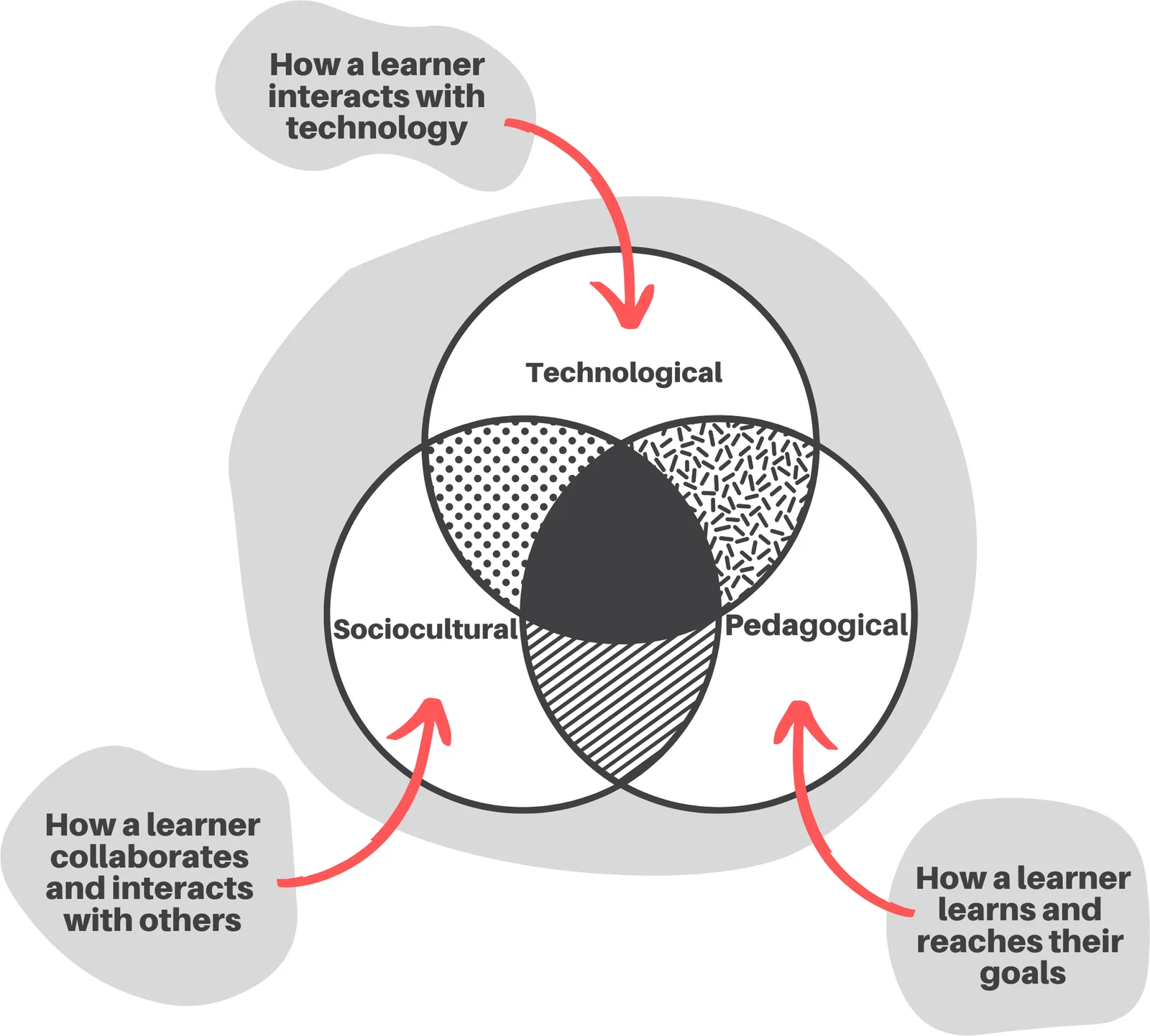
Sociotechnical-pedagogical usability framework reprinted with permission from Schmidt et al [40].

The sociotechnical-pedagogical framework addresses this gap by conceptualizing usability as a multimodal, emergent property of 3 interdependent dimensions: technological (eg, interface design and navigation), pedagogical (eg, learning activities, instructional clarity, and task relevance), and sociocultural (eg, cultural values, emotional responsiveness, and social norms), rather than as a property of the interface alone. These dimensions interact to shape users’ overall experience of a learning system (Figure 1). Importantly, the sociocultural dimension specifically recognizes that emotional responses, cultural backgrounds, and social contexts profoundly influence how individuals engage with and accept health learning technologies [40]. This integrated view is particularly relevant for PST mHealth interventions designed for populations with neurological or neurodevelopmental conditions, where sociocultural context and learning demands are inseparable from technological use.

To translate the sociotechnical-pedagogical framework into actionable usability assessment, Lu et al [41] developed the Comprehensive Assessment of Usability for Learning Technologies (CAUSLT), a validated instrument specifically designed to assess usability across all three sociotechnical-pedagogical dimensions. The CAUSLT was developed and empirically validated through multiple studies in learning contexts, demonstrating strong psychometric properties: exploratory factor analysis confirmed a coherent 3-factor structure aligned with the sociotechnical-pedagogical model (Kaiser-Meyer-Olkin=0.95; Bartlett test *P*<.001), with excellent overall internal consistency (Cronbach α=.95) and strong subscale reliability (technological α=.92; pedagogical α=.95; sociocultural α=.82) [41].

### Study Rationale and Aims

Despite increasing adoption of psychoeducational mHealth interventions, current usability evaluation practices remain technocentric, focused on interface functionality and user satisfaction, while largely neglecting pedagogical coherence and sociocultural responsiveness [31,33-35]. This limitation is particularly problematic for PST interventions, where learning is not incidental but essential to sustained self-management. The purpose of this collective case study, therefore, is to examine how usability is experienced and manifested across technological, pedagogical, and sociocultural dimensions within 3 psychoeducational PST mHealth interventions. Anchored in the sociotechnical-pedagogical framework [21] and its sociocultural extension [40], and operationalized using the CAUSLT evaluation instrument [41], this study identifies core design principles and recurring usability challenges, with the goal of informing more holistic, patient-centered approaches to digital health design.

### Research Questions

The following were the research questions (RQs):

1. How do participants perceive usability across technological, pedagogical, and sociocultural dimensions within mHealth PST interventions?
2. What recurring technological, pedagogical, and sociocultural usability challenges emerge across the three mHealth PST case studies?
3. What design principles can be derived from cross-case patterns in participants’ usability experiences?

## Methods

### Research Design Overview

This study used an exploratory, multimethod, collective case study design [42] in which 3 independently developed psychoeducational mHealth PST interventions were evaluated through structured think-aloud usability sessions. Each of the 3 cases (Epilepsy Journey 2.0, Survivor’s Journey, and electronic problem-solving training [ePST]) constituted an independent usability study with its own participant cohort, recruitment context, and data collection procedures. Findings from individual cases were subsequently examined together to identify recurring usability challenges and cross-case design principles. The multimethod approach integrated qualitative think-aloud usability data as the primary analytic source, and quantitative descriptive data from the CAUSLT instrument provided complementary, descriptive usability metrics for triangulation. Consistent with the exploratory and descriptive nature of this study, no inferential statistical tests were conducted across cases, and findings should be understood as preliminary.

### Intervention Descriptions

#### Overview

Three mHealth PST interventions evaluated in this study were independently developed for distinct clinical populations and are described below. While each intervention was developed separately, they share several core foundational features related to the structure and delivery of PST [4]. Each intervention implements the core ABCDE (Assess, Brainstorm, Consider and Choose, Develop, and Evaluate) problem-solving sequence, with ePST adding the F (Flex; ABCDEF [Assess, Brainstorm, Consider and Choose, Develop and Do, Evaluate, and Flex]) step. In addition, all interventions incorporate guided coaching components in certain conditions. Table 1 illustrates design overlaps across interventions.

**Table 1.** Common features across interventions.

Feature	Epilepsy Journey 2.0	Survivor’s Journey	ePST^a^
Clinical population	Adolescents with epilepsy	Adolescent and young adult survivors of brain tumors	Adults with a history of TBI^b^
PST^c^ Framework	ABCDE^d^	ABCDE	ABCDEF^e^ (additional Flex step)
Delivery Format	Mobile and web-based modules	Web-based modules	Mobile and web-based modules
Coaching Support	Therapist session	Therapist session	Coach session
Session Structure	10 themed virtual lands, each representing an instructional module	8 learning modules, with supplemental modules	5 learning modules, short lessons with problem-solving prompts
Learning Focus	Executive functioning support	Coping and daily functioning	Independent problem-solving and self-efficacy

a ePST: electronic problem-solving training.

b TBI: traumatic brain injury.

c PST: problem-solving training.

d ABCDE: Assess, Brainstorm, Consider and Choose, Develop, and Evaluate.

e ABCDEF: Assess, Brainstorm, Consider and Choose, Develop and Do, Evaluate, and Flex.

#### Case 1: Epilepsy Journey 2.0

**Figure 2.**
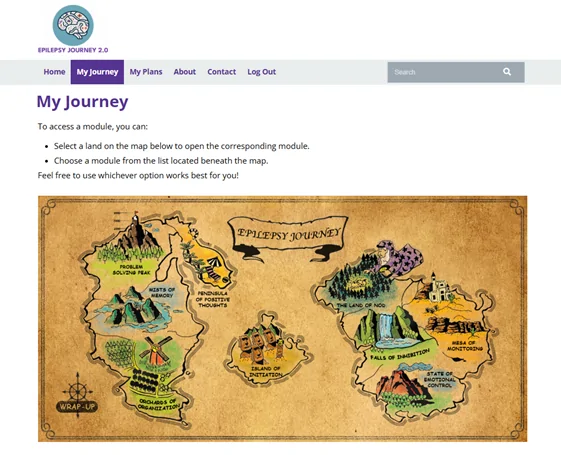
Epilepsy Journey 2.0 interface.

Epilepsy Journey 2.0 is a web-based, mHealth PST intervention designed to support adolescents with epilepsy who experience executive functioning difficulties (Figure 2). The intervention includes multimedia instruction, short interactive tasks, and scaffolded guidance to support self-regulation, planning, and problem-solving skills. Instruction includes a 5-step problem-solving sequence using the “ABCDE” mnemonic. Content is organized using a “journey” metaphor, in which users progress through 10 themed virtual lands, each representing an instructional module related to executive functioning [43,44].

#### Case 2: Survivor’s Journey

**Figure 3.**
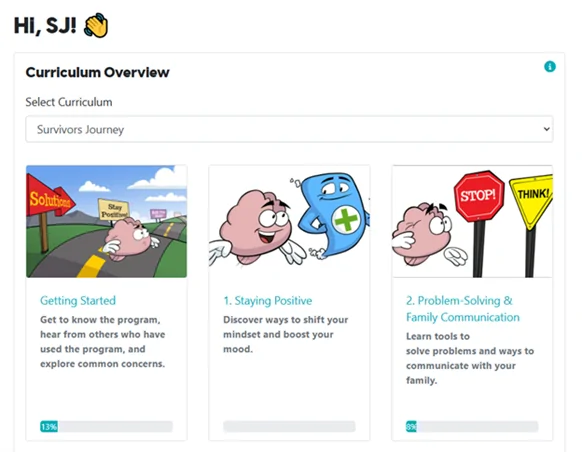
Survivor’s Journey interface.

Survivor’s Journey is a web-based psychosocial intervention designed to provide problem-solving therapy and coping skills training for adolescent and young adult survivors of brain tumors (Figure 3). It delivers PST-based coping and adjustment strategies through interactive multimedia lessons, therapist-supported sessions, and tailored problem-solving activities. The problem-solving sequence also adopts the “ABCDE” mnemonic. Modules focus on managing late effects, emotional regulation, fatigue, and everyday functional challenges among adolescent and young adult survivors of pediatric brain tumors. Instruction is structured through a stepwise problem-solving framework and emphasizes real-world applicability using developmentally appropriate scenarios. The platform has undergone feasibility and preliminary efficacy testing [45].

#### Case 3: ePST

**Figure 4.**
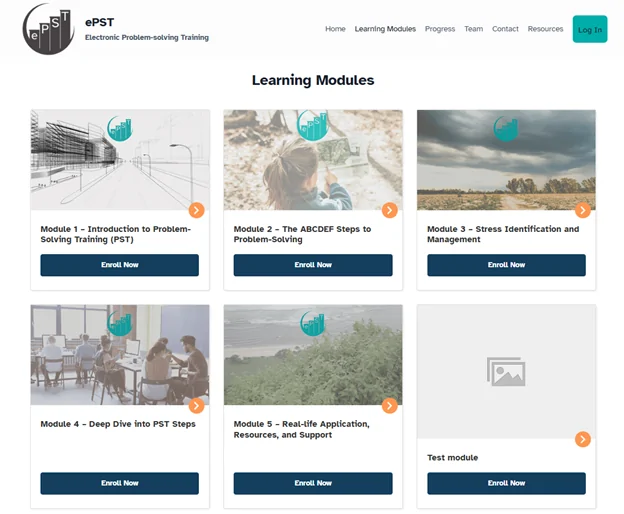
ePST learning environment. ABCDEF: Assess, Brainstorm, Consider and Choose, Develop and Do, Evaluate, and Flex; ePST: electronic problem-solving training; PST: problem-solving training.

ePST is a self-guided mHealth PST intervention for adults with a history of traumatic brain injury (TBI) with sustained loss of consciousness (characterized as “severe”; Figure 4). ePST builds on a traditional problem-solving framework by adapting it into an interactive, digital format to enhance accessibility and autonomy for users. The program teaches a 6-step problem-solving sequence using the “ABCDEF” mnemonic. Content is delivered through short lessons, interactive decision points, personal examples, and initial guidance from a coach to support users in applying PST strategies independently and at their own pace. Recent development work has described its person-centered adaptation process and community-based participatory design components [46,47].

### Participant Characteristics

**Figure 5.**
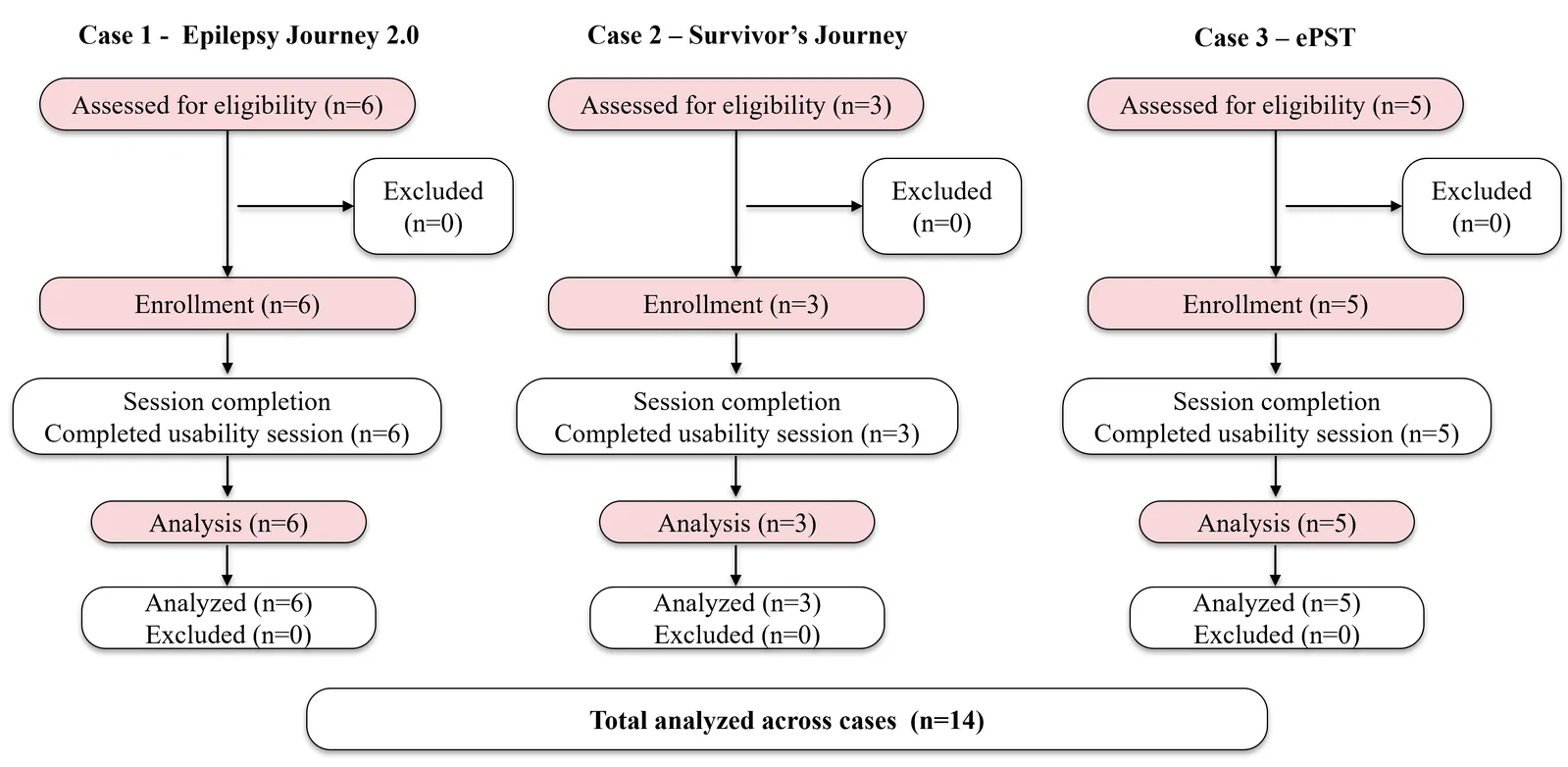
Participants flowchart. ePST: electronic problem-solving training.

A total of 14 participants were enrolled across the 3 usability studies. Table 2 presents participant characteristics by case, while Figure 5 presents the participant flowchart.

**Table 2.** Participants’ characteristics and recruitment parameters.

Case	mHealth intervention	Participants, n	Recruitment parameters
1	Epilepsy Journey 2.0	6 (*M*=4, *F*=2)	Adolescents living with epilepsy who were able to navigate a digital platform and provide verbal feedback.
2	Survivor’s Journey	3 (*M*=1, *F*=2)	Young adult survivors of brain tumors who were able to participate in remote usability testing and provide feedback on usability, navigation, and support for learning and coping goals.
3	ePST^a^	5 (*M*=2, *F*=3)	Adults who had sustained a severe TBI^b^ 11 to 20 years prior and who were able to participate in a virtual session independently

a ePST: electronic problem-solving training.

b TBI: traumatic brain injury.

### Inclusion and Exclusion Criteria

Participants were recruited from the intended user populations for each respective intervention. Inclusion and exclusion criteria were defined at the case level. For case 1 (Epilepsy Journey 2.0), eligibility included adolescents with a confirmed clinical diagnosis of epilepsy who were able to access and navigate a web-based digital platform and provide verbal feedback during a usability session. For case 2 (Survivor’s Journey), eligibility included young adults aged between 18 and 30 years with a documented history of pediatric brain tumor survivorship who were able to participate independently in a remote usability session and provide verbal feedback on platform navigation, learning content, and problem-solving supports. For case 3 (ePST), eligibility included adults with a documented history of severe TBI occurring 11 to 20 years before participation who were able to engage independently in a virtual session and had access to a personal smartphone or computer with internet connectivity.

Across the 3 cases, participants were excluded if they were unable to complete at least one assigned module during the usability session, could not provide verbal feedback, or lacked the technical access required to participate. No additional clinical exclusion criteria were applied beyond the diagnostic and functional requirements specified for each case in the preceding paragraph.

### Sampling Procedures and Participant Recruitment

Purposive sampling was used across all 3 case studies, with participants selected for their direct experiential relevance to the intended user populations of each mHealth PST intervention. This approach was appropriate given the exploratory nature of the study and the goal of obtaining usability feedback from individuals with lived experience relevant to the interventions being evaluated [48].

Case 1 participants (n=6) were recruited through an existing advisory board established as part of the Epilepsy Journey 2.0 development process at Cincinnati Children’s Hospital Medical Center (CCHMC). The advisory board comprised adolescents and young adults with epilepsy, their caregivers, health care providers, and educators. Usability participants were drawn from the adolescent member subset of this board. Case 2 participants (n=3) were recruited through clinical and research networks at CCHMC, including the pediatric neuro-oncology program. Eligible young adult survivors of brain tumor were identified by the clinical research team and invited to participate via direct outreach. Case 3 participants (n=5) were recruited through clinical and research settings affiliated with UT (University of Texas) Health Houston and TIRR (The Institute for Rehabilitation and Research) Memorial Hermann. Recruitment materials were shared within these medical center settings, and individuals who expressed interest were screened for eligibility based on TBI history and functional capacity to participate independently.

The sample sizes were determined by the independent protocols, recruitment timelines, and feasibility constraints of each respective usability study. In formative usability research, relatively small samples are commonly used for problem discovery, with foundational studies showing that the first 4 to 5 participants often identify a substantial proportion of major usability problems [49-51]. At the same time, later work has cautioned that 5 users should not be treated as a universal rule, because larger samples improve stability and increase the proportion of problems detected, particularly when systems or user groups are more heterogeneous [52]. Consistent with this broader literature, recommendations for usability studies in health-related digital systems commonly place formative samples in the range of approximately 5 to 10 participants, with larger samples warranted when interface complexity or user diversity is greater. On this basis, the sample sizes for case 1 (n=6) and case 3 (n=5) were considered appropriate for identifying prominent usability issues within their respective intervention contexts, whereas the smaller sample in case 2 (n=3) is interpreted more cautiously and its findings are presented as preliminary.

### Measures and Instruments

Quantitative data were collected from two sources: (1) a brief preassessment questionnaire evaluating participants’ general technology comfort and familiarity with digital learning platforms, and (2) the CAUSLT scale. For the technology comfort assessment, participants responded to a five-item Likert-type technology comfort survey. It comprised five items which assessed: (1) frequency of eHealth app use; (2) general comfort with digital technology; (3) comfort using digital health or educational tools specifically; (4) confidence navigating new mHealth platforms; and (5) prior experience with interventions similar to the one being tested. Each item was rated on a 5-point scale (1=not at all comfortable/experienced to 5=extremely comfortable/experienced). Responses were summarized using descriptive statistics across all participants. For CAUSLT, the instrument comprises 20 items rated on a 5-point Likert scale (1=strongly disagree to 5=strongly agree), organized into 3 subscales aligned with the sociotechnical-pedagogical framework [41]. Item scores were averaged within each subscale and converted to a 0‐100 standardized scale to facilitate interpretation and cross-subscale comparison.

### Ethical Considerations

The 3 studies reported in this paper were reviewed and approved by the appropriate institutional review boards (IRBs). The Epilepsy Journey 2.0 and Survivor’s Journey studies were approved by the CCHMC IRB (Federalwide Assurance #00002988). Epilepsy Journey 2.0 recruited an advisory board of adolescents/adults, caregivers, health care providers, and teachers. For the purpose of this study, only adolescents who completed usability testing were included. Due to the advisory board nature, only an agreement form was signed, and participation implied consent, as they were considered consultants, not participants. Survivor’s Journey followed the same institutional review requirements and received CCHMC IRB approval under protocol 2024‐0546. The ePST usability study was reviewed by the University of Georgia Human Research Protection Program (IRB #00009943) and was deemed exempt under federal guidelines.

All participants provided written informed consent before data collection. For remote sessions, consent was obtained electronically using Qualtrics; for in-person sessions, consent was documented via approved parental permission, assent, or adult consent forms, as required. Consent documents described study procedures, audio or video recording, risks and benefits, voluntary participation, HIPAA (Health Insurance Portability and Accountability Act) authorization where applicable, and data handling. All data were deidentified before analysis and stored on encrypted institutional servers with access restricted to authorized research personnel. Raw recordings and master linking files will be destroyed at the conclusion of each study. Participants in the ePST study received US $25 per session. Advisory board members in Epilepsy Journey 2.0 were compensated US $50 per advisory board meeting they participated in (up to 3 meetings). Participants in the Survivor’s Journey usability study received US $50. No identifying information is presented in this paper or supplementary materials.

### Data Collection Procedures

#### Overview

Usability testing was conducted across the 3 mHealth PST interventions between June 2024 and July 2025. Across all studies, participants completed a structured think-aloud session in which they interacted with selected modules of their assigned intervention, followed by a postsession CAUSLT survey and a presession technology comfort survey. Sessions were conducted remotely via Zoom, except for 1 in-person session for Epilepsy Journey 2.0. A standardized “think-aloud” protocol was used, including facilitator-led introductions, verbal consent, verbal reflection prompts, and observational note-taking. All sessions were audio- and video-recorded and transcribed for analysis.

#### Case Study 1: Epilepsy Journey 2.0

Conducted in June and August 2024 (n=6; 5 remotely and 1 in person), this study evaluated ease of use, interface design, content delivery, and interactive features. Participants explored the platform, completed 1 assigned module focused on memory or problem-solving, and provided feedback through verbal protocols.

#### Case Study 2: Survivor’s Journey

Conducted remotely in June and July 2025 (n=3), this study assessed navigation, functionality, and problem-solving support features. Participants explored the platform by completing account sign-up and landing-page tasks, followed by working through a learning module and using the problem-solving tools.

#### Case Study 3: ePST

Conducted remotely in July 2024 (n=5), this study evaluated the introductory and advanced modules focused on the problem-solving strategies. Each participant completed 2 assigned lessons addressing the full “ABCDEF” problem-solving strategy. Participants accessed the platform via personal smartphones (1 participant used a personal computer) and were prompted to reflect on both ease of use and learning support.

### Data Analysis

#### Quantitative Data Analysis

Quantitative data were analyzed using descriptive statistics appropriate to the exploratory and small-sample nature of each case study. No inferential statistical tests were conducted, and no α level was set. CAUSLT item scores (rated 1‐5) were converted to a standardized 0‐100 scale by averaging item responses within each subscale and rescaling proportionally ((mean−1)/4×100). Case-level subscale means and overall usability means were calculated by averaging participant-level standardized scores within each intervention. Technology comfort survey data were analyzed using descriptive statistics (frequencies and percentages) and reported at the case level to contextualize participants’ baseline familiarity with digital tools. Regarding missing data, complete CAUSLT and technology comfort survey data were obtained from all 14 enrolled participants. No item-level missing data were identified in any case.

#### Qualitative Data Analysis

The qualitative component of the study followed established usability testing procedures rather than thematic coding approaches. Session recordings, think-aloud verbalizations, and observer field notes served as the primary qualitative data sources. A semistructured, task-based usability protocol guided each session, during which participants completed a series of predefined tasks while thinking aloud and sharing their screens. Tasks targeted both technological usability (eg, navigation and multimedia interaction) and pedagogical usability (eg, clarity of content and instructional structure).

Transcripts generated from Zoom’s automated transcription tool were reviewed for accuracy and used alongside field notes to identify instances where participants encountered navigation difficulties, misunderstandings, or breakdowns in task flow. Two trained members of the research team independently reviewed and discussed each usability session to document observed usability barriers and to verify that identified issues were grounded in direct participant behavior rather than interpretive coding. The team collaboratively reviewed usability issues and assigned severity ratings using the Nielsen 5-point severity framework [53], which categorizes usability problems as: 0=not a usability problem; 1=cosmetic problem only (need not be fixed unless time allows); 2=minor usability problem (low priority to fix); 3=major usability problem (important to fix, high priority); and 4=usability catastrophe (imperative to fix before product release). Disparities in ratings were discussed until consensus was reached. In the case that consensus could not be reached within the research team, the faculty advisor was consulted. This only occurred once, with an issue related to the loading time of 1 video. Representative usability issues and corresponding severity ratings are provided in Multimedia Appendix 1. All team members who participated in usability data analysis received training in applying the Nielsen severity scale as part of the research group’s standard usability research training procedures, including review of criteria definitions, worked examples, and calibration exercises before the study. Efficiency-related data, such as time on task and error rates, were extracted from session recordings and incorporated into the analysis. Findings were summarized in structured usability reports for each intervention.

## Results

### Overview

This section presents findings from the exploratory usability evaluation across the 3 case studies. No cross-case statistical comparisons were made. Quantitative findings are reported descriptively and interpreted within the context of each case. First, participants’ technology comfort levels are summarized to contextualize subsequent usability findings. Next, CAUSLT results are reported for overall usability and for the technological, pedagogical, and sociocultural dimensions aligned with the sociotechnical-pedagogical framework. Finally, we highlight recurring usability challenges and corresponding design principles that emerged in relation to these dimensions.

### RQ1: How Do Participants Perceive Usability Across Technological, Pedagogical, and Sociocultural Dimensions Within mHealth PST Interventions?

#### Participants’ Technology Comfort Levels

Participants reported varied levels of technology comfort across the 3 interventions. In Epilepsy Journey 2.0, half of the participants (3/6, 50%) identified as experienced technology users; in ePST, 3 of 5 (60%) participants did so; and in Survivor’s Journey, two-thirds (2/3, 66.7%) of participants also identified as experienced users. Most participants reported feeling comfortable using technology both in general and for health or educational purposes, including 83.3% (5/6) of participants in Epilepsy Journey 2.0, 100% (3/3) of participants in Survivor’s Journey, and 60% (3/5) of participants in ePST. Prior experience with eHealth or digital health tools varied, with the majority in each intervention indicating occasional use. Confidence using technology independently ranged from slightly confident to very confident across all interventions. Detailed case level technology comfort responses are provided in Multimedia Appendix 2.

#### CAUSLT Results

##### Overall Usability

**Figure 6.**
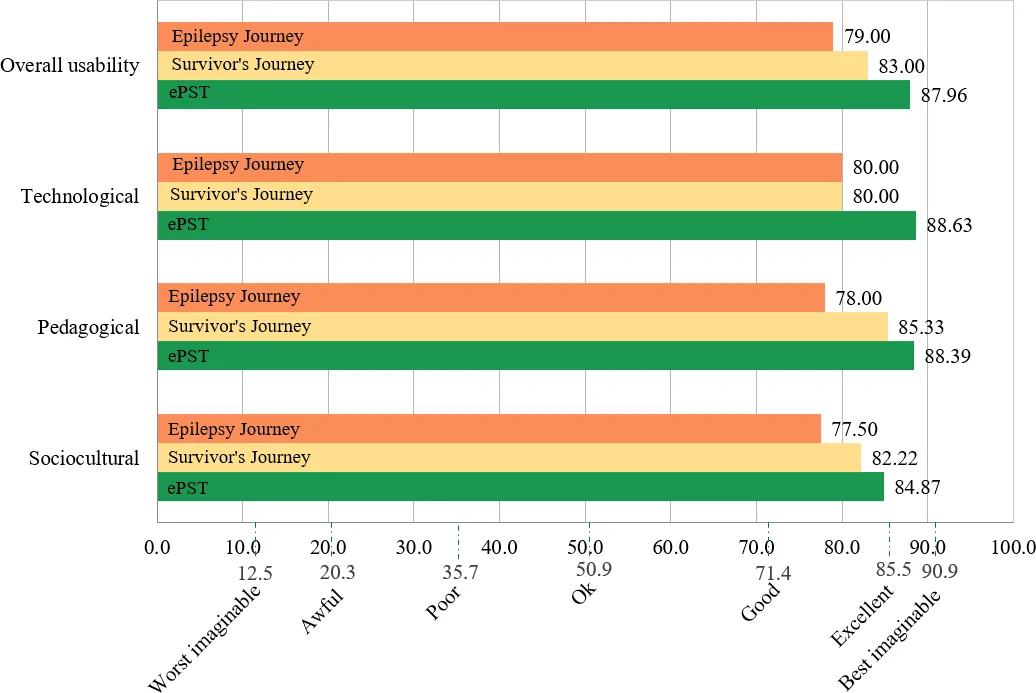
Average Comprehensive Assessment of Usability for Learning Technologies scores across the 3 mHealth (mobile health) interventions based on sociotechnical-pedagogical dimensions. ePST: electronic problem-solving training.

Figure 6 displays average CAUSLT scores across the 3 mHealth PST interventions. Scores are reported on a standardized 0 to 100 scale. All 3 interventions received overall mean scores in the upper range of the scale, indicating generally high participant agreement that the interventions exhibited usability characteristics across the measured dimensions. ePST received the highest overall mean score (87.96, SD 13.73), reflecting consistently high participant agreement across technological, pedagogical, and sociocultural dimensions. Survivor’s Journey and Epilepsy Journey 2.0 received an overall mean score of 83.00 (SD 7.70) and 79.00 (SD 12.75), respectively, reflecting similarly strong overall agreement.

##### Technological Usability

**Figure 7.**
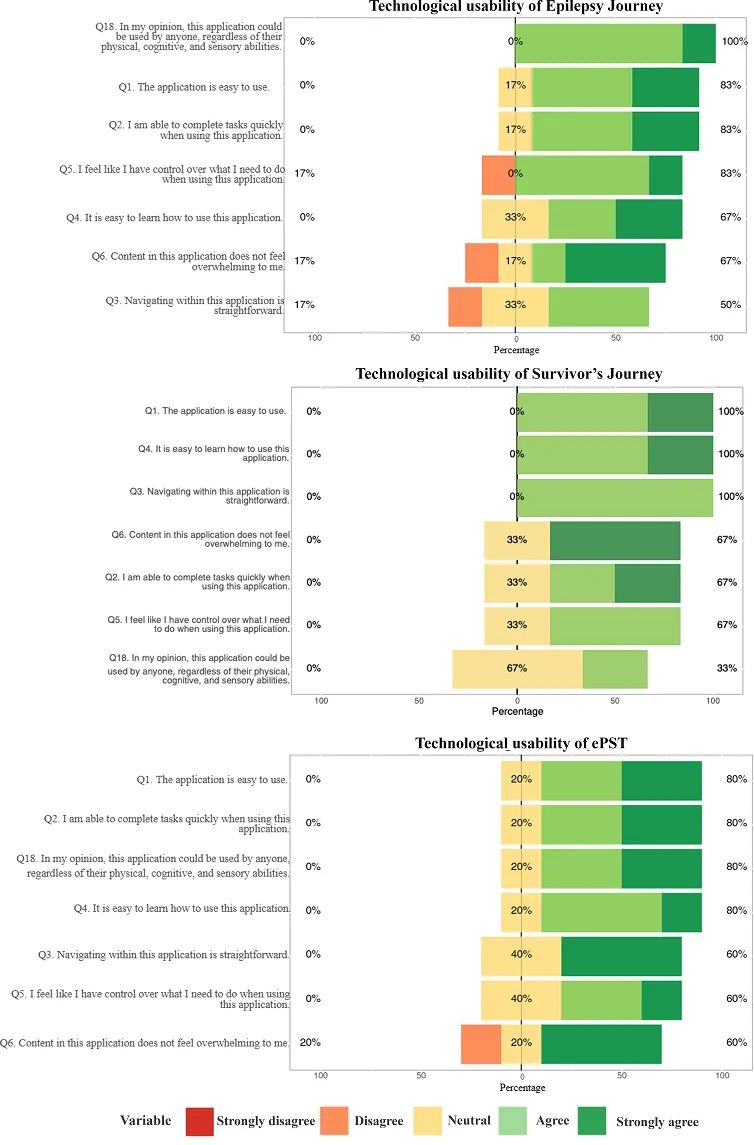
Technology usability of 3 interventions. ePST: electronic problem-solving training.

Technological usability was assessed using the 7 technological CAUSLT items (Figure 7). ePST received the highest technological mean scores, reflecting strong participant agreement across items related to ease of navigation and functional clarity. Additionally, Epilepsy Journey 2.0 and Survivor’s Journey both received technological mean scores in the upper portion of the scale, with somewhat lower agreement on items related to navigation clarity and user control. These items reflected areas in which participants indicated difficulty understanding interface structure or completing tasks without assistance, suggesting room for technological improvement in both cases.

##### Pedagogical Usability

**Figure 8.**
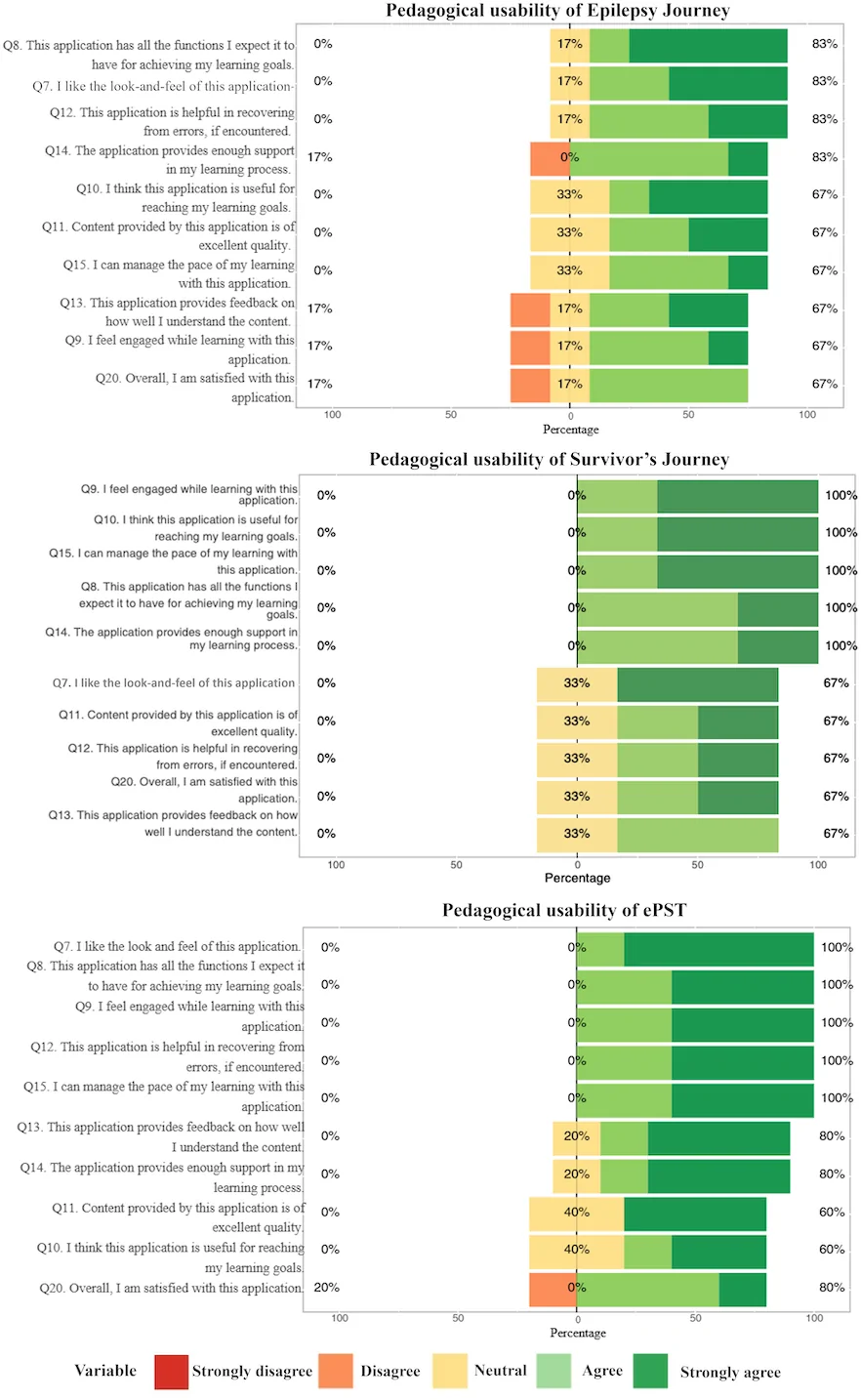
Pedagogical usability of three interventions. ePST: electronic problem-solving training.

Pedagogical usability results are shown in Figure 8. ePST received high mean scores across items reflecting instructional support, content clarity, and learning goal alignment. Epilepsy Journey 2.0 received high mean scores, particularly for items related to engagement and satisfaction, with slightly lower agreement on items concerning instructional clarity and guidance. Survivor’s Journey showed consistently strong pedagogical usability, with high mean scores for learning goals, engagement, and pacing, and slightly lower ratings on items related to instructional support and content quality.

##### Sociocultural Usability

**Figure 9.**
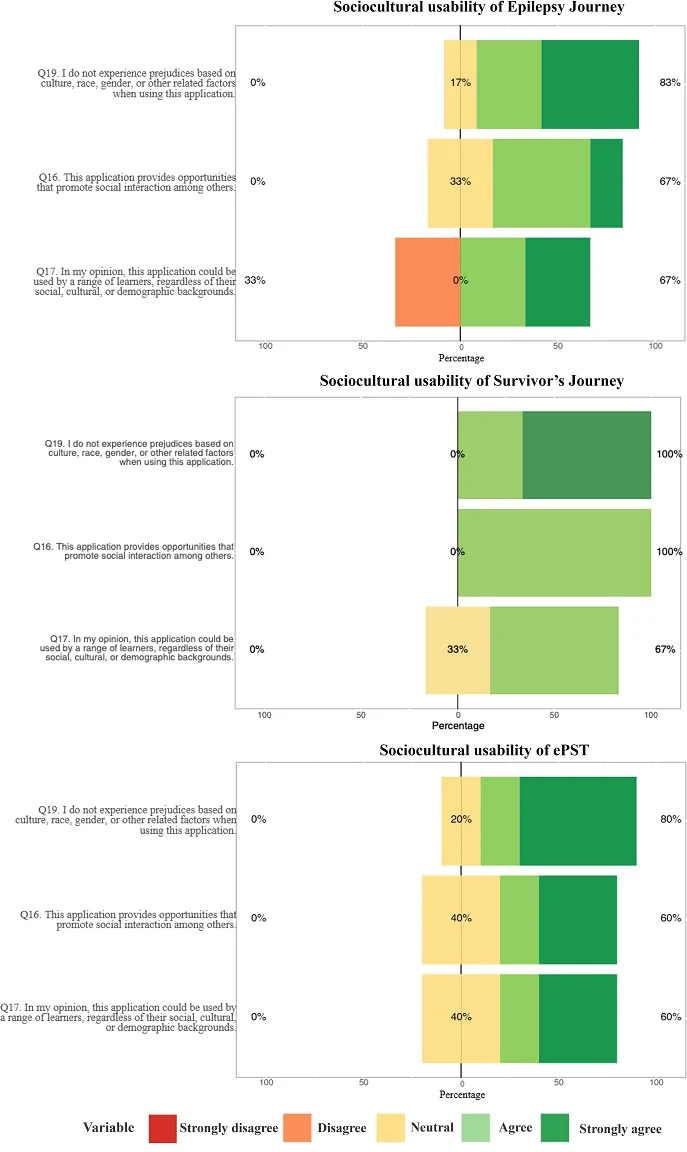
Sociocultural usability of 3 interventions. ePST: electronic problem-solving training.

Sociocultural usability was assessed using 3 CAUSLT items focused on inclusivity, lack of prejudice, and opportunities for social interaction (Figure 9). ePST received high mean sociocultural scores, with slightly lower agreement on whether the intervention could be used by a wide range of learners. Survivor’s Journey also demonstrated predominantly excellent sociocultural usability, particularly for perceptions of safety and opportunities for social interaction, with somewhat lower mean scores on items related to accommodating diverse users. Epilepsy Journey 2.0 received somewhat lower mean sociocultural scores relative to the other 2 cases, with greater variability across items and more neutral responses regarding social interaction and inclusivity. These scores indicate that participants experienced fewer supports for resolving errors or receiving corrective guidance in this intervention.

### RQ2: What Recurring Technological, Pedagogical, and Sociocultural Usability Challenges Emerge Across the 3 mHealth PST Case Studies?

#### Overview

The think-aloud sessions and field notes across all 3 case studies yielded a set of preliminary usability challenges that appeared with some consistency across interventions, participants, and sociotechnical-pedagogical dimensions. Representative usability challenges and their associated Nielsen severity ratings are summarized in Multimedia Appendix 1. These challenges are organized below by dimension.

#### Technological Usability Challenges

##### Overview

Participants across all 3 interventions identified recurring technological difficulties, including unclear navigation structures, technical performance issues, limited mobile compatibility and readability, confusing progress indicators, and problematic visual design and layout.

##### Inconsistent and Confusing Navigation Structures

Participants frequently reported uncertainty about how to proceed within modules. In Epilepsy Journey 2.0, a participant stated, “I found it unclear how to start and navigate through the modules and was confused about what button to select at the end of a module.” In ePST, a participant (Pe3) described unexpected behavior when using arrow-based navigation. Similarly, all participants in Survivor’s Journey reported navigation confusion due to misleading labels and screen indicators that did not accurately match where they were in the modules. For example, 1 participant (Ps2) noted that “the system sometimes displays ‘Session Content’ even when I am still on the Start page, and I’m not sure whether clicking Save or Next will complete a task or simply store my progress.”

##### Poor Mobile Responsiveness and Technical Glitches

Participants noted difficulties when accessing the interventions on smartphones, including slow loading and multimedia glitches. In ePST, a participant (Pe5) remarked, “The video didn’t load properly, and it caused confusion for me.” In Survivor’s Journey, participants encountered system glitches that disrupted smooth task completion. These included the “site tour” repeatedly appearing even after completion and certain features such as the AI-supported Refine button being positioned in ways that made them effectively hidden or difficult to locate.

##### Lack of Clear Progress Tracking

Participants in Epilepsy Journey 2.0 and Survivor’s Journey reported uncertainty about task completion status, stating that it was not always clear whether activities were finished or how far along they were within a module.

### Pedagogical Usability Challenges

#### Overview

Participants reported several pedagogical issues related to how the interventions supported learning, cognitive engagement, and instructional flow. Key issues included instructional ambiguity, sequencing gaps, information overload, and limited feedback.

#### Instructional Ambiguity and Sequencing Gaps

Participants across interventions described uncertainty about intended next steps. In Epilepsy Journey 2.0, a participant (Pj5) stated, “I wasn’t sure whether I was supposed to read or click.” ePST participants reported difficulty recalling and following multistep instructions after completing videos. Survivor’s Journey users described similar uncertainty regarding task order and inconsistent message design.

#### Dense Content Affecting Working Memory

Participants in Epilepsy Journey 2.0 and Survivor’s Journey noted that some pages contained dense text or tightly condensed content that was challenging to process. A participant in Survivor’s Journey (Ps1) explicitly struggled with poor message design regarding text hierarchy and reported confusion caused by redundant and repetitive messages, while Epilepsy Journey 2.0 participants commented on sections being “too wordy.”

#### Limited Feedback and Progress Cues

Across interventions, several participants expressed uncertainty about whether their actions had been recorded or whether they had completed tasks. Epilepsy Journey 2.0 users noted difficulty determining module completion status, and ePST participants occasionally interpreted badges as indicators of progress. Survivor’s Journey participants also reported uncertainty about overall progress, as they could not distinguish between the progress bar and actual lesson completion.

### Sociocultural Usability Challenges

#### Overview

Participants identified sociocultural concerns related to emotional sensitivity, inclusivity, representational authenticity, and error recovery.

#### Content Sensitivity and Emotional Impact

In Epilepsy Journey 2.0, a participant (Pj4) expressed concern about a flashing background and questioned, “if it was intended to cause seizures.” In ePST, a participant reported that the orange color scheme was intimidating, and a motivational quote felt misaligned with their expectations as a user with a brain injury. In Survivor’s Journey, 1 participant (Ps1) expressed that the website’s name did not clearly communicate that the intervention was designed for survivors of pediatric brain tumors, which lessened her confidence in its relevance and purpose.

#### Accessibility and Inclusivity Beyond Technical Features

Participants noted that mobile display challenges affected readability and ease of use for individuals relying exclusively on smartphones. These concerns extended beyond technological accessibility, reflecting broader needs for inclusive visual, cognitive, and layout design.

#### Relevance and Relatability of Content and Narratives

In ePST, a participant (Pe5) commented that “someone in the lesson does not appear to be brain injured,” raising questions about the perceived visual authenticity of character portrayals. In contrast, participants in Epilepsy Journey 2.0 noted that characters and testimonials felt relatable.

#### Lack of Support for Error Recovery and Comprehension Feedback

Participants across cases expressed interest in clearer explanations when answers were incorrect or when navigation errors occurred. For example, a participant in Epilepsy Journey 2.0 (Pj4) stated that understanding “why an answer was wrong would be helpful for learning and retention.”

### RQ3: What Design Principles Can Be Derived From Cross-Case Patterns in Participants’ Usability Experiences?

Cross-case analysis of participants’ usability experiences resulted in a set of preliminary design considerations that appeared with directional consistency across the 3 mHealth PST interventions. These considerations were organized into technological, pedagogical, and sociocultural categories to align with the multidimensional structure of the CAUSLT and the broader sociotechnical-pedagogical framework. Table 3 presents the resulting design principles, each linked to observed usability challenges and participant-reported needs.

**Table 3.** Usability-driven design principles that emerged across 3 mHealth^a^ PST^b^ interventions.

Dimension and design principle	Description
Technological	
Intuitive, consistent navigation	Use clear labeling, standardize button behavior, and ensure directional cues are predictable to reduce confusion and improve wayfinding.
Mobile-first design	Optimize layout, font size, multimedia compatibility, and interaction design specifically for smartphone use, particularly for users with no access to a desktop.
Transparent progress tracking	Provide clear, visual indicators of task and module completion to help users track their learning and reduce frustration.
In-platform troubleshooting support	Include tooltips, guided walkthroughs, or pop-ups to help users recover from errors and reduce reliance on external help.
Pedagogical	
Clear instructional guidance	Use explicit instructions, consistent “next step” cues, and scaffolded transitions to guide learners through the intervention without ambiguity.
Reduce cognitive overload	Break up long text blocks, use visual hierarchy, and incorporate multimedia to improve comprehension and decrease fatigue.
Context-sensitive feedback and completion cues	Provide immediate, relevant feedback and clear visual indicators of progress or task completion to reinforce engagement and comprehension.
Use narrative and interactive learning strategies	Incorporate relatable characters, scenarios, or gamified interactions to enhance learner connection and motivation.
Sociocultural	
Safety-conscious and empathetic design	Avoid triggering visuals (eg, flashing), intimidating color schemes, or emotionally insensitive language; test content with end users.
Universal accessibility and flexibility	Design with inclusive features such as adjustable font size, high contrast, and alternative inputs to accommodate diverse users and contexts.
Authentic, relatable content	Include stories, testimonials, and characters that reflect the lived experiences, language, and identities of the target user population.
Provide feedback for error recovery and reflection	Offer explanatory feedback on incorrect answers and missed actions to foster user confidence, learning, and self-efficacy.

a mHealth: mobile health.

b PST: problem-solving training.

## Discussion

### Principal Findings

This exploratory study aimed to examine how usability manifests across technological, pedagogical, and sociocultural dimensions within 3 mHealth PST interventions and to identify recurring issues within and across cases that influence patient learning and engagement. Using the sociotechnical-pedagogical framework as an analytic lens, the findings reveal how these dimensions interact to shape user experience and highlight observations regarding challenges within each case. Within and across cases, quantitative CAUSLT findings indicated generally high perceived usability, alongside meaningful variability across dimensions and interventions that highlighted specific areas for improvement. Complementing these findings, qualitative think-aloud data and observational field notes revealed a recurring set of usability challenges spanning navigation ambiguity, limitations in mobile responsiveness, instructional uncertainty, cognitive overload, insufficient feedback, and condition-specific sociocultural concerns. Despite differences in clinical populations and intervention design, these challenges exhibited notable directional consistency across cases. Synthesis of observations within each case further yielded 12 preliminary design principles organized along sociotechnical-pedagogical dimensions and grounded in observed user behaviors. In the following sections, we discuss key insights associated with each RQ. We conclude by reviewing the study’s limitations and outlining implications for future mHealth learning design and evaluation.

### RQ1: Participants’ Perceptions of Usability Across Technological, Pedagogical, and Sociocultural Dimensions

Across all 3 interventions, participants’ perceptions suggested that technological, pedagogical, and sociocultural usability operate as mutually reinforcing dimensions rather than isolated components. Interventions with strong interface clarity, such as ePST and Epilepsy Journey 2.0, appeared to provide a smoother entry point into the learning experience, but participants’ comments showed that interface success alone did not determine overall usability. Even when navigation was described as intuitive, uncertainties about task flow, expectations, and progress frequently shaped how users interpreted the system’s coherence. This pattern suggests that technological usability is necessary, but not sufficient, in mHealth PST contexts, where learning (not just interaction) drives engagement.

Conversely, the Survivor’s Journey case study offered a particularly illustrative, though preliminary, window into the consequences of pedagogical and sociocultural misalignment. Despite receiving relatively strong technological scores, participants in that intervention encountered what Nielsen [53] would classify as “usability catastrophes” in the pedagogical domain, where the pedagogical structure failed to communicate progress accurately, leaving participants unsure whether they had completed learning objectives or inadvertently skipped content. These challenges illustrate that users evaluate usability not solely by whether the system works, but also by whether it supports comprehension, reduces cognitive load, and acknowledges their context of use, in alignment with research suggesting that patients interpret usability through the lens of learning demands, emotional states, and everyday constraints (not just interface performance) [21,54,55].

Taken together, these preliminary observations support the view that usability in learning-oriented mHealth interventions is most productively understood as a relational property, one that materializes at the intersection of how the technology performs, how the instruction guides, and how the content speaks to participants’ experiential realities [21,40]. This integrated perspective is consistent with broader arguments in health informatics that narrowly technocentric usability evaluation fails to capture the dimensions of the user experience most consequential for clinical engagement and behavior change [31-34].

### RQ2: Recurring Usability Challenges Across Technological, Pedagogical, and Sociocultural Dimensions

Within each intervention case, usability challenges reflected potential misalignments across technological, pedagogical, and sociocultural dimensions. Rather than isolated interface flaws, participants’ experiences within each case suggested deeper tensions relative to how users attempted to navigate, learn from, and make sense of the interventions. Within individual cases, when 1 dimension falters, it appeared to trigger or amplify challenges in the others, suggesting an entangled nature of usability in mHealth PST contexts [40]. For example, in Survivor’s Journey, participants’ difficulties distinguishing between progress storage and task completion (a technological navigation issue) undermined their ability to determine whether learning objectives had been met, resulting in pedagogical uncertainty and disrupted task flow. Similarly, in Epilepsy Journey 2.0, the absence of clear module completion indicators and ambiguous navigation cues left participants uncertain not only about their location within the intervention but also about whether they had fully engaged with core PST components, reflecting cascading pedagogical consequences of technological signaling gaps.

Technological challenges, such as inconsistent navigation pathways, limited mobile responsiveness, and unstable multimedia performance, frequently disrupted participants’ ability to orient themselves within the interventions. Even when individual screens appeared well designed, unclear directional cues or difficulty returning to previous sections undermined participants’ sense of control and progress. These issues point to a need to consider technological usability not as surface-level interface performance but as a foundational condition that can enable or constrain participants’ learning. Indeed, when navigation or responsiveness became unpredictable, participants struggled to maintain focus on problem-solving tasks, indicating that technological clarity is a prerequisite for cognitive engagement. In this case sample, the participants with brain tumors (n=3) indicated a preference for tablets or desktops over smartphones. While this preliminary observation in a limited sample suggests that mobile-only design may introduce accessibility burdens for certain users with specific neurological conditions, validation in larger samples is needed before drawing broader conclusions about device preferences.

Pedagogical challenges emerged when instructional sequences, task expectations, or content density failed to align with participants’ cognitive needs. Ambiguity about what to do next, difficulty following multistep processes, and the burden of overly dense or text-heavy material created avoidable cognitive load. These challenges were not simply presentation issues. Instead, they reflect gaps in how systems guide users through the problem-solving process. Even when the interface itself was functional, pedagogical misalignment disrupted participants’ ability to interpret, retain, and apply the PST framework. Further, sociocultural challenges shaped participants’ experiences by influencing how understood and represented they felt while using a given intervention. Elements that might seem minor from a design perspective, such as color schemes, character portrayals, or emotionally neutral phrasing, sometimes carried unintended emotional weight for participants with neurological conditions. For example, when examples felt inauthentic or insensitive, participants questioned the intervention’s relevance and legitimacy. Participants’ judgments about usability were shaped as much by cultural resonance, emotional tone, and instructional clarity as by the mechanics of the interface itself.

Collectively, these recurring challenges demonstrate that usability breakdowns are rarely confined to a single point of failure. Difficulties with navigation, comprehension, or emotional resonance often co-occurred and reinforced one another, illustrating the interdependence of sociotechnical-pedagogical dimensions and underscoring the utility of the sociotechnical-pedagogical framework for identifying these interactions. The systemic perspective offered by the sociotechnical-pedagogical framework underscores why mHealth PST interventions require integrated, context-sensitive design approaches able to anticipate the cognitive and emotional realities of diverse patient populations.

### RQ3: Design Principles Derived From Cross-Case Usability Patterns

Building on the recurring usability challenges identified across the 3 interventions, the third RQ focused on how these patterns translate into actionable design principles for mHealth PST environments. Findings from this exploratory study revealed that effective usability is not the outcome of isolated interface improvements, but the result of design decisions that simultaneously support technological clarity, pedagogical coherence, and sociocultural resonance. The preliminary design principles derived illustrate how these dimensions interact to shape patient experience and highlight that usability emerges from the alignment of these layers rather than from any single dimension in isolation.

Across cases, technological design principles emphasize the need for intuitive navigation pathways, mobile-first responsiveness, transparent progress indicators, and embedded troubleshooting support. These principles suggest that ease of use in mHealth PST interventions involves more than functional stability. It requires interfaces that anticipate learners’ cognitive flow, device constraints, and need to recover quickly from errors. Participant experiences showed that technical clarity enables deeper engagement with problem-solving content, whereas even small inconsistencies in navigation or responsiveness disrupt orientation and confidence. Thus, technological usability functions as the infrastructural layer that supports, rather than competes with, the learning process.

Pedagogical design principles emphasized instructional clarity, reduced cognitive load, context-sensitive feedback, and the use of narrative or interactive elements to support comprehension. Interventions in which instructions were explicit, sequences were well-paced, and content was visually organized allowed participants to focus on the “ABCDE” or “ABCDEF” steps of PST rather than the mechanics of navigating the system. Conversely, ambiguous instructions or dense content increased cognitive effort and weakened participants’ ability to internalize core problem-solving strategies. Such patterns demonstrate the centrality of pedagogical usability to learning-oriented mHealth design, in which the quality of instructional scaffolding can directly shape users’ performance and perceptions of system usability.

Finally, sociocultural design principles extended usability beyond technological and pedagogical domains and into the areas of sociocultural authenticity and inclusive representation. Participant perceptions demonstrated that issues such as color choice, narrative tone, and representational accuracy can meaningfully shape trust, comfort, and identification with the content. When these elements aligned with participants’ lived experiences, responses suggested that participants perceived the intervention as supportive and credible, whereas when they did not, participants perceived the system as less usable, even if the technology and underlying pedagogy were otherwise sound. This suggests that sociocultural usability mediates how technological and pedagogical decisions are interpreted and underscores the need for co-design practices that meaningfully involve target populations through design and development phases [56].

As a whole, the design principles derived from RQ3 reveal that usability is best understood as a relational construct that materializes when technological, pedagogical, and sociocultural dimensions are coherently integrated. The sociotechnical-pedagogical framework proved particularly effective for identifying where misalignments occurred and how they could be addressed through design. This integrated view offers a path forward for developing digital health PST interventions that are not only functional but also instructionally sound and culturally responsive.

### Limitations

Several important limitations should be considered when interpreting these findings. First, and most critical, the sample sizes across all 3 cases were small, and the sample for case 2 (Survivor’s Journey; n=3) falls below the commonly cited 5-participant threshold for formative usability testing [50]. The small sample hence limits transferability and precludes inferential cross-case conclusions. Second, demographic data, including race, ethnicity, gender, age, and educational background, were not systematically collected as part of these usability evaluations. This limits the ability to examine how these dimensions interact with condition-specific sociocultural usability experiences. While the sociotechnical-pedagogical framework’s sociocultural dimension in this study was operationalized through participants’ condition-specific lived experience (as described in the Methods section), it is recognized that demographic factors may intersect with and amplify condition-specific experiences in ways that this study could not detect. Future studies should systematically collect and report demographic data to enable a more complete examination of sociocultural usability. Third, usability testing was predominantly conducted in controlled, virtual environments, potentially affecting participants’ natural interactions with the platforms. Real-world usage scenarios might reveal additional usability issues not captured in this structured testing context. Fourth, participant familiarity with technology varied significantly across interventions, which may have influenced usability perceptions independently of the interventions’ inherent design. However, it is notable that the intervention with the highest overall usability also included participants with the lowest average confidence using technology; it may be that those with more technology familiarity also have higher expectations and are more critical than those with less familiarity (rather than those with less familiarity simply struggling more). Finally, this study’s primary reliance on observational data without longitudinal follow-up restricts the ability to assess sustained usability perceptions and real-world intervention effectiveness.

### Comparison With Prior Work

The preliminary findings of this exploratory study align with and extend prior research that argues for multidimensional approaches to usability in digital health. Consistent with earlier work, technological elements such as navigation clarity, visual readability, and mobile responsiveness remain foundational determinants of user satisfaction and engagement [40,55,56]. However, this study demonstrates that technological usability alone cannot fully account for how individuals evaluate or experience digital health PST interventions. Instead, technological features become meaningful only when aligned with the pedagogical and sociocultural demands of learning and self-management. This perspective aligns with emerging critiques in the mHealth literature that evaluations focusing only on technological factors tend to overlook the bigger picture of how users interpret and make sense of an intervention as a learning environment [57,58].

Pedagogically, this study supports previous arguments by Jahnke et al [21] and others advocating for the incorporation of instructional clarity, cognitive load management, and meaningful feedback into usability design. However, the present findings extend this literature by showing empirically how instructional misalignment often underlies perceived usability failures, emphasizing that interface efficiency must be evaluated alongside how effectively a system teaches, guides, and motivates its patients [21,41]. Socioculturally, the results resonate with scholars [40,59] who emphasize emotional safety, cultural sensitivity, and inclusivity as vital to mHealth engagement. Yet, this study advances prior work by demonstrating that sociocultural usability functions as a mediating layer, shaping how technological and pedagogical design decisions are perceived, trusted, and adopted by diverse users. Empathetic design and authentic representation thus emerge as central to achieving sustained participation and emotional connection [47,56].

More broadly, these findings contribute to a growing body of literature calling for multidimensional usability evaluation in digital health. Reviews of current practice have consistently identified a reliance on single-dimensional instruments, most commonly the SUS, across the field, with little attention to pedagogical or sociocultural dimensions [31-35]. The present study provides a concrete, case-level demonstration of what such evaluations miss: in 2 of the 3 cases, CAUSLT subscale scores revealed variation across sociotechnical-pedagogical dimensions that would not have been visible with a single overall usability score, and think-aloud data revealed condition-specific sociocultural concerns absent from the technological usability narrative. The CAUSLT, as operationalized in this study, proved useful for surfacing these multidimensional patterns, though, as noted in the Methods section, no validated benchmark classifications for the CAUSLT exist yet, and the instrument’s psychometric performance in clinical populations beyond those represented here remains to be established [41]. Future research should adopt longitudinal designs, include more diverse populations, and be conducted in real-world clinical settings to examine how technological, pedagogical, and sociocultural factors dynamically interact to shape usability in psychoeducational mHealth interventions. Such work is needed to clarify how multidimensional usability influences engagement, learning processes, and behavioral health outcomes over time.

### Implications and Future Directions

The preliminary findings of this exploratory study present several implications for mHealth PST design, evaluation practice, and future research, implications that extend beyond the specific interventions examined here. At the most fundamental level, these findings provide initial evidence that the prevailing practice of evaluating psychoeducational mHealth tools primarily on the basis of interface-level usability metrics is likely to produce an incomplete and potentially misleading picture of whether an intervention is ready for clinical deployment. An mHealth PST tool that scores well on the SUS or similar instruments, but whose instructional scaffolding generates cognitive overload, whose feedback mechanisms fail to support error recovery, or whose characters and scenarios feel inauthentic to the target population, may be perceived as technically usable while failing to deliver meaningful psychoeducational benefit. The CAUSLT, operationalized within the sociotechnical-pedagogical framework, offers a more complete evaluation approach precisely because it surfaces these multidimensional gaps. Broader adoption of multidimensional evaluation tools in the formative development of digital health interventions could improve the alignment between usability, learning, and clinical effectiveness, contributing to stronger engagement, adherence, and ultimately better health outcomes for patients using PST mHealth tools.

For intervention developers and health care teams, the 12 preliminary design principles derived from this study offer a starting framework for building multidimensional usability into the design process from the outset, rather than addressing usability deficits reactively after development. Mobile-first design, explicit instructional scaffolding, context-sensitive feedback, and co-design with condition-specific communities are not complex or expensive design choices; they are design commitments that reflect a genuine prioritization of the user’s learning experience. The growing literature on community-based participatory approaches in digital health design [56] suggests that these commitments are most effectively implemented when the target population is involved as a genuine partner in the design process rather than as a late-stage test user.

For researchers, these findings suggest several high-priority directions for future work. First, the 12 design principles identified here require prospective empirical testing to determine whether implementing them improves usability, learning, engagement, and clinical outcomes in PST mHealth contexts. Second, the CAUSLT requires further psychometric validation within clinical populations with neurological and neurodevelopmental conditions, including the development of population-specific descriptive norms that would enable more meaningful score interpretation than is currently possible. Third, longitudinal usability research is needed to understand how usability experiences at first interaction predict sustained engagement, treatment completion, and behavioral health outcomes over time, a critical but currently understudied link in the mHealth effectiveness chain. Fourth, future studies should use larger, more diverse samples within each condition-specific population and should systematically collect demographic data to enable examination of intersecting sociocultural dimensions of usability experience.

In sum, this exploratory study provides preliminary evidence that usability in psychoeducational mHealth PST interventions is a multidimensional construct shaped by the coherent integration of technological, pedagogical, and sociocultural design decisions. When these dimensions are aligned, the intervention creates conditions for meaningful engagement with PST content; when they diverge, the result is not merely user inconvenience but a potential disruption of the very learning process that the intervention is designed to support. Advancing the field requires evaluation practices and design approaches that take this multidimensionality seriously, and this study offers an initial empirical foundation from which that advancement can proceed.

## Supplementary material

10.2196/90379 Multimedia Appendix 1 Usability issues identified across all 3 cases with Nielsen severity ratings.

10.2196/90379 Multimedia Appendix 2 Participant comfort level with teComfort Level with Technology.

## Abbreviations

ABCDE: Assess, Brainstorm, Consider and Choose, Develop, and Evaluate
ABCDEF: Assess, Brainstorm, Consider and Choose, Develop and Do, Evaluate, and Flex
CAUSLT: Comprehensive Assessment of Usability for Learning Technologies
CCHMC: Cincinnati Children’s Hospital Medical Center
ePST: electronic problem-solving training
HIPAA: Health Insurance Portability and Accountability Act
IRB: institutional review board
mHealth: mobile health
PST: problem-solving training
RQ: research question
SUS: System Usability Scale
TBI: traumatic brain injury
TIRR: The Institute for Rehabilitation and Research
UT: University of Texas
