# Supplementary material for: Usability Across 3 mHealth Problem-Solving Training Interventions for Diverse Neurodevelopmental and Neurological Populations: Multicase Usability Evaluation
Source: JMIR Hum Factors. 2026 Sep 9;13:e90379. doi: 10.2196/90379 (PMC13557615; doi:10.2196/90379)
Supplement: Supplementary file 1 [file humanfactors-v13-e90379-s001.docx]

| **STP**  **Dimension** | **Usability Challenges** | **Case** | **Specific Issue Description** | **Participants Affected** | **Nielsen Severity (0-4)** | **Recommendation** |
| --- | --- | --- | --- | --- | --- | --- |
| **Technological** | Inconsistent and confusing navigation structures | Epilepsy Journey 2.0 | Unclear navigation pathways; participants unsure how to proceed within modules. | 5/6 (83%) | 3 | Provide clear instructions and visual navigation cues. |
|  |  | Epilepsy Journey 2.0 | Unintended navigation loops leaving users unsure if they finished a lesson. | 2/6 (33%) | 4 | Fix "Mark complete" page logic and implement robust progression tracking. |
|  |  | Survivor’s Journey | Inconsistent information architecture; confusing site tour that repeats despite completion. | 2/3 (67%) | 2 | Fix site tour logic and standardize navigation patterns. |
|  |  | Survivor’s Journey | Breadcrumb navigation indicated incorrect module locations. | 1/3 (33%) | 3 | Clarify or relabel breadcrumbs and ensure accurate color contrast. |
|  |  | ePST | Forward navigation arrow not visible in the mobile version of Storyline. | 1/5 (20%) | 3 | Update instructions to provide mobile navigation directions. |
|  |  | ePST | Inconsistent button design and positioning across screens. | 2/5 (40%) | 2 | Standardize button design and positioning across all screens. |
|  | Poor mobile responsiveness and technical glitches | Epilepsy Journey 2.0 | Severe mobile responsiveness issues; overlapping text, unreadable fonts, and videos failing to load. | 3/6 (50%) | 4 | Adopt a mobile-first design approach and perform rigorous QA on mobile. |
|  |  | Epilepsy Journey 2.0 | Scrolling issues causing the screen to jump unexpectedly. | 2/6 (33%) | 3 | Fix scrolling behavior and break long pages into smaller sections. |
|  |  | Survivor’s Journey | Hidden or difficult-to-locate features due to layout (e.g., AI-supported Refine button). | 1/3 (33%) | 3 | Improve feature visibility and placement. |
|  |  | ePST | Content visibility issues requiring zoom/orientation changes; responsive layout problems. | 2/5 (40%) | 3 | Improve responsive layout so orientation changes are not necessary. |
|  |  | ePST | Layout and responsiveness issues with landscape mode; screen changes unexpectedly. | 1/5 (20%) | 3 | Improve layout stability across phone orientations. |
|  |  | ePST | Video playback failures; content not displaying (audio-only playback). | 1/5 (20%) | 4 | Fix video encoding for low-bandwidth scenarios; test across conditions. |
|  | Lack of clear progress tracking | Epilepsy Journey 2.0 | Lack of clear progress tracking; participants uncertain about module completion status. | 4/6 (67%) | 4 | Implement comprehensive progress bars and completion messages. |
|  |  | Survivor’s Journey | Unclear navigation between progress storage and task completion; misleading status indicators. | 3/3 (100%) | 3 | Clarify distinction between “Save” and task completion; fix status display. |
|  |  | Survivor’s Journey | Incomplete and inaccurate progress bar synchronization. | 3/3 (100%) | 3 | Redesign progress bar to synchronize accurately with user activity. |
|  |  | ePST | Confusion about the completion status of badges (e.g., greyed-out badges). | 1/5 (20%) | 2 | Provide clearer indicators for badge completion status. |
| **Pedagogical** | Instructional ambiguity and sequencing gaps | Epilepsy Journey 2.0 | Participants confused about expected actions (read vs. interact). | 5/6 (83%) | 3 | Provide clear, concise instructions for each task with visual cues. |
|  |  | Survivor’s Journey | Instructional ambiguity about required tasks and sequencing. | 3/3 (100%) | 3 | Clarify task requirements and sequencing logic. |
|  |  | Survivor’s Journey | Lack of clarity on adding multiple solutions (Singular "Solution" label). | 2/3 (67%) | 3 | Change label to “Solutions” and provide clear cues for multiple entries. |
|  |  | ePST | Insufficient task clarity and completion requirements messaging. | 4/5 (80%) | 3 | Add clear messaging about completion requirements. |
|  |  | ePST | Incorrect navigation instructions provided in the text. | 3/5 (60%) | 3 | Review and correct all instructional text to accurately reflect required actions. |
|  | Dense content affecting working memory | Epilepsy Journey 2.0 | Text-heavy content without sufficient multimedia engagement. | 3/6 (50%) | 2 | Balance text with more interactive elements and multimedia. |
|  |  | Survivor’s Journey | Insufficient learning scaffolding; content density overwhelming for some users. | 1/3 (33%) | 3 | Add stepwise scaffolding; break content into smaller chunks. |
|  |  | ePST | Long sentence structures increasing cognitive load; required reading aloud. | 2/5 (40%) | 3 | Reduce sentence lengths, split them up, and add text-to-speech audio. |
|  | Limited feedback and progress cues | Epilepsy Journey 2.0 | Inconsistent feedback mechanisms; limited corrective guidance when errors made. | 5/6 (83%) | 3 | Implement immediate, meaningful feedback on assessments. |
|  |  | Survivor’s Journey | Ambiguity regarding evaluation scores. | 2/3 (67%) | 2 | Ensure evaluation scores are clearly explained with a summary checklist. |
|  |  | ePST | Feedback given on incorrect answers in assessments seemed unclear. | 1/5 (20%) | 2 | Review and refine assessment feedback to ensure it is helpful. |
| **Sociocultural** | Content sensitivity and emotional impact | Epilepsy Journey 2.0 | Flashing background animations posing a seizure risk. | 1/6 (17%) | 4 | Remove flashing backgrounds and use static, non-distracting images. |
|  |  | ePST | Sensitivity of motivational quote content; insensitive phrasing toward individuals with TBI. | 1/5 (20%) | 2 | Rephrase quote to be more empathetic and inclusive. |
|  |  | ePST | "Intimidating" bright orange color scheme. | 1/5 (20%) | 2 | Consider using a softer, more neutral color palette suitable for brain-injured users. |
|  | Relevance and relatability of content and narratives | Survivor’s Journey | Website name fails to convey focus on pediatric brain tumor survivors. | 1/3 (33%) | 1 | Revise the platform name to be more specific to the target audience. |
|  |  | Survivor’s Journey | Limited accommodation for diverse learning needs; content tailored to narrow user profile. | 2/3 (67%) | 2 | Increase accessibility features and representation. |
