# Supplementary material for: Usability Across 3 mHealth Problem-Solving Training Interventions for Diverse Neurodevelopmental and Neurological Populations: Multicase Usability Evaluation
Source: JMIR Hum Factors. 2026 Sep 9;13:e90379. doi: 10.2196/90379 (PMC13557615; doi:10.2196/90379)
Supplement: Supplementary file 2 [file humanfactors-v13-e90379-s002.doc]

| **Question** | **Scale** | **Epilepsy Journey 2.0** | **Survivor’s Journey** | **ePST** |
| --- | --- | --- | --- | --- |
| **Q1** = How would you describe yourself as a technology user? | 1. Expert  2. Experienced  3. Capable  4. Novice | 30%  50%  20%  0% | 0%  66.6%  33.3%  0% | 0%  66.6%  33.3%  0% |
| **Q2** = How comfortable do you feel using technology in general (like smartphones, tablets, computers)? | 1. Very Comfortable  2. Comfortable  3. Uncomfortable  4. Very Uncomfortable | 16.6%  83.3%  0%  0% | 100%  0%  0%  0% | 33.3%  66.6%  0%  0% |
| **Q3** = How frequently have you used digital tools or e-health platforms for health management before? | 1. Often 2. Sometimes 3. Rarely 4. Never | 16.7%  50%  16.7%  16.7% | 0%  66.6%  0%  33.3% | 0%  80%  0%  20% |
| **Q4** = How would you rate your comfort level with using technology for educational or health-related purposes? | 1. Very Comfortable  2. Comfortable  3. Uncomfortable  4. Very Uncomfortable | 33.3%  66.6%  0%  0% | 100%  0%  0%  0% | 40%  60%  0%  0% |
| **Q5** = How confident are you that you will be able to use this courseware without additional help? | 1. Very Confident 2. Confident 3. Slightly Confident 4. Not Confident | 16.6%  33.3%  50%  0% | %  100%  0%  0% | 0%  100%  0%  0% |
